# Can Distalisation and Lateralisation Shoulder Angles in Reverse Arthroplasty Interfere with the Functional Results in Patients with Rotator Cuff Arthropathy?

**DOI:** 10.1055/s-0044-1779609

**Published:** 2024-03-21

**Authors:** Tamara Dainotto, Diego Gómez, Glenda Ernst

**Affiliations:** 1 Departamento deOrtopedia e Traumatologia, Hospital Británico de Buenos Aires, Buenos Aires, Argentina; 2 Conselho Científico, Hospital Británico de Buenos Aires, Buenos Aires, Argentina

**Keywords:** arthroplasty, rotator cuff, shoulder

## Abstract

**Objective:** To evaluate the influence of radiographic values on clinical and functional results in patients treated with reverse arthroplasty for rotator cuff arthropathy (RCA) using a lateralized design.

**Methods:** A retrospective analysis was performed. Patient demographics were recorded, as well as preoperative and postoperative range of motion. Function was calculated using the Constant-Murley score both before and after the procedure. Pre and postoperative anteroposterior and axial radiographs of the affected shoulder were analysed. In the preoperative images, the following was calculated: acromiohumeral distance (AHD) and lateral humeral offset (LHO). Postoperative measurements included: AHD, LHO, distalization shoulder angle (DSA) and lateralisation shoulder angle (LSA). Linear regression and quadratic regression analysis was performed to determine their degree of association with final functional outcomes. By applying a quadratic regression analysis and ROC curves, the cut-off values were determined with respect to the above-mentioned angles and the positive predictive value was calculated.

**Results:** The greater anterior elevation (AE) ranges were found with DSA between 40-45° and LSA among 80°- 90°, while better ABD was observed with LSA of 90-100°. Preoperative AHD was correlated to RE (r _s_ :0.47; p:0.049). Postoperative AHD was found to be in a directly proportional relationship with AE (r _s_ :0.49; p:0.03). Postoperative ABD showed an inverse linear regression with preoperative AHD (r _s_ : -0.44, p:0.047). LSA and DSA were inversely related.

**Conclusion:** We found that a DSA between 40-45° and a LSA of 80-100° could lead to better range of motion regarding AE and ABD in patients with rotator cuff arthropathy treated with RSA.

## Introduction

The original design of the shoulder inverted arthroplasty (IA) by Grammont in 1985 for the treatment of rotator cuff arthropathy (RCA), consisted of medializing and distalizing the center of rotation of the glenohumeral joint to enhance the deltoid lever arm and thus achieve a higher range of anterior elevation (AE) and abduction (ABD). 1 2 3 These implants were associated with some specific complications, such as generation of scapular notches and neurological damage 1 2 3 that decreased thanks to the introduction of lateralized reversed shoulder arthroplasties (RSA) which also achieved a lower incidence of prosthetic dislocation and a greater range in rotations. 4

Controversy exists regarding the ideal implant positioning to maximise range of motion and reduce the risk of complications. The debate is also present in relation to radiographic values and their influence on postoperative results. 5 Numerous authors have tried to determine the ideal degree of lateralisation and distalisation in RSA, even though the methods have been considered too demanding for daily clinical practice. 5 6 7 8 Shoulder distalisation and lateralisation angles described by Boutsiadis et al., 8 which describe the humeral position in relation to the scapula, could represent reproducible tools and determinants of clinical results.

The aim of this study was to identify the radiographic values and to evaluate their influence on results in patients treated with RSA for RCA using a lateralized design with 135° of humeral inclination.

## Methods

A retrospective analysis between January 2018 and January 2020 was performed. This article was approved by the Ethics Committee.

### Patient Selection

The exclusion criteria were patients treated with medialized prostheses or with humeral inclination of 145° or 155°; concomitant presence of humeral head or glenoid fractures; absence of preoperative radiographs; revision surgeries; insufficient follow-up and neuromuscular diseases. Included patients had a minimum follow up of 12 months and were treated for rotator cuff arthropathy (Hamada ≥ 3) with a lateralized prosthesis with 135° humeral inclination. They were 18 years old or more and had functioning deltoid.

### Surgical Technique

The Arrow® lateralized prosthesis (FH Orthopaedics, Mulhouse, France) was used.

With the patient in a beach chair position, under plexus anaesthesia, a deltopectoral approach is performed, with complete insertional tenotomy of the subscapularis and section of the joint capsule. The humeral head is dislocated in maximum external rotation and the humeral osteotomy is performed with 20° of retroversion and 135° of inclination. Subsequently, the humeral canal is worked with increasing rasps until a sensation of cortical friction is obtained. The largest calibre rasp used is left in place to protect the proximal humerus during the glenoid tempo.

When performing the glenoid exposure, osteophytes and degenerative labrum are resected, optimising the view of the articular surface. Then, the articular cartilage is milled, preserving as much bone stock as possible. The implantation of the metaglene is carried out by projecting a lower inclination of 10° with a neutral version. Definitive fixation is done with two 5.5 mm compression screws. This system allows the use of 36, 39 or 42 mm glenospheres.

The definitive humeral component is placed uncemented, as long as the metaphyseal bone quality allows it. The size of the final polyethylene insert is then selected which as well as the size of the glenosphere, are decided according to the intraoperative deltoid tension and stability of the implant.

All patients are immobilised for 30 days with a Velpeau-type sling, starting passive mobility exercises one week after surgery and rehabilitation four weeks later.

### Clinical Evaluation

Preoperative range of motion was analysed, as well as the postoperative range in the last control performed. Active mobility was evaluated in degrees with a goniometer in AE, ER1, ABD and internal rotation (IR) with the hand on the back, recording the segment reached by the thumb as the maximum level according to the description of Greene and Heckman, 9 and then punctuated as Levy et al. 10

Deltoid's function was defined by the treating surgeon, according to the Daniel's motor scale, considering deltoid functioning when equating with a value of M5. 11

Function was calculated using the adapted Constant-Murley score for Argentinian population. 12

### Radiological Evaluation

Anteroposterior (AP) and axial radiographs of the affected shoulder, both preoperative and postoperative, were used. Considering the importance of correct positioning and radiological technique, all images were carried out by the same personnel with the same fluoroscope.

The measurements were determined in the AP projection by two researchers unaware of the clinical results with the Synapse 3d® software (Fujifilm Healthcare®), considering the interobserver average. Interobserver agreement was calculated by intraclass correlation coefficient (ICC). The power of ICC was considered when the agreement was > 0.8. It was almost perfect in all measurements, as demonstrated in Table 1 .

**Table 1:** Measurements of radiographic values expressed in mean and standard deviation (SD)

	Mean (SD)	Rango	ICC	ICC 95% CI
**Age (years)**	72 (±7.1)	61–84		
**Pre AHD**	7.27 mm (±4.1)	1–16.2	0.96	(0.81–0.99)
**Pre LHO**	12.56 mm (±5.8)	3–23.5	0.98	(0.95–0.99)
**Pop AHD**	25.6 mm (±8.3)	12–46	0.97	(0.93–0.98)
**Pop LHO**	18.6 mm (±7.3)	2–28.2	0.95	(0.89–0.97)
**DSA**	43.2° (±6.8)	30–60	0.88	(0.53–0.96)
**LSA**	92.5° (±10.1)	80–115	0.82	(0.62–0.92)
**CMS**	69.9 (±7.8)	47–83		

Intraclass correlation coefficient for each measurement and its range are displayed.

Abbreviations: CI, confidence interval; CM, Constant-Murley score; DSA pop, distalisation shoulder angle; ICC, intraclass correlation coefficient; LSA, lateralisation shoulder angle; popAHD, postoperative acromiohumeral distance; pop LHO, postoperative lateral humeral offset; PreAHD, preoperative acromiohumeral distance; preLHO, preoperative lateral humeral offset.

**Fig. 1:**
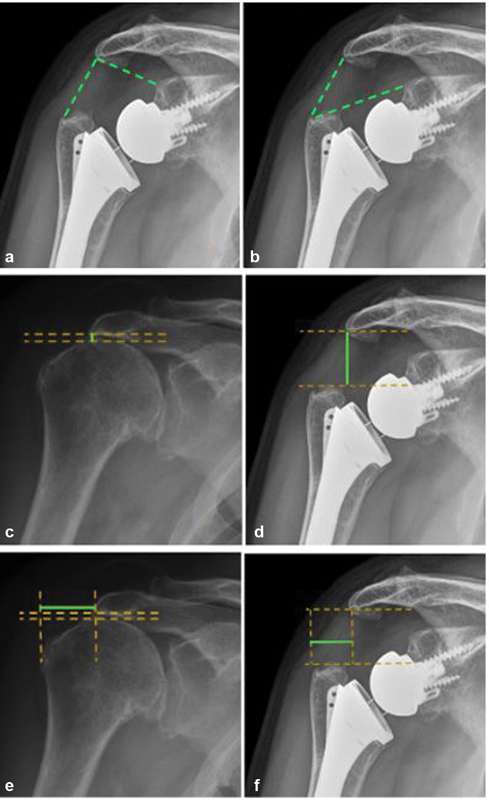
Measurement descriptions. a) lateralisation shoulder angle; b) distalistion shoulder angle; c) preoperative acromiohumeral distance; d): postoperative acromiohumeral distance; e) preoperative lateral humeral offset; f) postoperative lateral humeral offset.

In the preoperative images, the following were calculated: acromiohumeral distance (AHD) and lateral humeral offset (LHO). Postoperative measurements included: AHD, LHO, DSA and LSA ( Fig. 1 ).

- For LSA ( Fig. 1A ), taking three landmarks: superior border of the glenoid tubercle, the most lateral border of the acromion and the most lateral border of the greater tuberosity. A line will be drawn joining the superior glenoid tubercle with the most lateral border of the acromion. A second line connects this last point with the lateral border of the greater tuberosity. The angle between these two lines corresponds to the LSA.
- For DSA ( Fig. 1B ), considering the superior border of the glenoid tubercle, the most lateral border of the acromion and the most superior border of the greater tuberosity, will be calculated by drawing a line from the most lateral border of the acromion to the superior glenoid tubercle and from this, another line to the most superior border of the greater tuberosity. The angle between these two lines corresponds to the DSA.
- AHD is measured by calculating the perpendicular distance between the most lateral portion of the acromion and a parallel line to the superior border of the greater tuberosity ( Fig. 1C-D ).
- To calculate the LHO, the distance from the AHD line to a projection to the most lateral edge of the greater tuberosity is drawn ( Fig. 1E-F ).

The Hamada and Fukuda classification 13 was used to determine the degree of glenohumeral osteoarthritis.

### Statistical Analysis

Continuous descriptive variables were expressed as mean, standard deviation and range. Qualitative variables were expressed as percentages. Linear regression analysis was performed to determine the degree of association between preoperative and postoperative angles and mobility. A quadratic regression analysis was applied and the area under the ROC curve AUC-ROC was used. We used cut-point value as the value whose sensitivity and specificity were closer to value of the AUC-ROC and absolute difference between sensitivity and specificity value was minimum. The cut-off values were determined with respect to the angles DSA and LSA. Graph Pad Prism 8.02 software and MedCalc12.0 were used.

## Results

### Patients

**Fig. 2:**
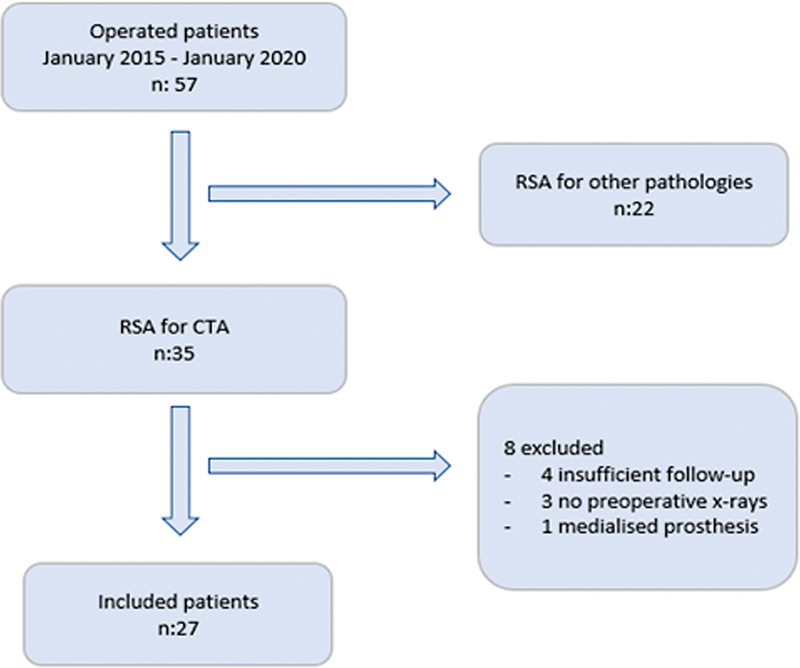
Flowchart of included and excluded patients. RSA: reverse shoulder arthroplasty; CTA; cuff tear arthropathy.

Out of 57 patients who went under RSA, 35 were diagnosed with RCA. Eight of them were excluded; it was impossible to obtain the radiographies of 3 patients, 4 of them had not enough follow-up, and a medialised prosthesis was used in the remaining one. ( Fig. 2 ). Twenty-seven patients with a mean age of 72.0 ± 7.1 were studied. (77.7% women, n: 21-20 right shoulders). We did not analise range of motion (ROM) with regard to the size of the glenosphere, which was 36 mm in 24 cases and 39 mm in three cases. The time to follow-up survey was 19.3 ± 6.9 months postoperatively. Mean LSA was 92.5° (85-115°) and that of DSA was 43.2° (30-60°). The mean values of radiographic measurements are described in Table 1 as well as standard deviation, intraclass correlation coefficient and coefficient confidence intervals. Preoperative and postoperative mobility is registered in Table 2 , showing significative differences between values except for the abduction (p:0.56)

**Table 2:** Preoperative and postoperative values regarding mobility

	Preoperative Mean (SD)	Postoperative Mean (SD)	p
**Anterior elevation**	90.21° (39.8°)	131.2° (32°)	0.0002
**External rotation**	12.3° (19.2)	35.2° (16.8°)	<0.0001
**Internal rotation**	L4-L5 (3.8) SD 2.2	L1-L3 (5.5) SD 2.4	0.013
**Abduction**	68.7° (31.9°)	87.1° (28.2°)	0.56

### Correlation Analysis

A directly proportional association was found between preoperative AHD and ER and RI (r _s_ : 0.47 and r _s_ :0.44, respectively); while there was a negative Pearson correlation with the ABD (r _s_ : -0.44). Between postoperative AHD and AE, a direct proportional Spearman association was observed (r _s_ :0.49). As evidenced in Table 3 , no association was observed between the rest of studied angles.

**Table 3:** Correlation analysis

**PreAHD**	***r***	***p***
AE	0.0004	0.99
ER	0.47	0.049
ABD	−0.44	0.047
IR	0.44	0.046
CM	0.36	0.07
**PreLHO**	***r***	***p***
AE	−0.0025	0.9
ER	0.17	0.42
ABD	−0.04	0.86
IR	−0.43	0.04
CM	0.12	0.6
**AHD**	***r***	***p***
AE	0.13	0.5
ER	−0.05	0.8
ABD	25	0.9
IR	0.06	0.8
CM	0.06	0.7
**ALH**	***r***	***p***
AE	−8	0.7
ER	−0.14	0.49
ABD	0.25	0.2
IR	−0.24	0.28
CM	−0.3	0.13
**PopADH**	***r***	***P***
AE	0.49	0.03
ER	0.32	0.11
ABD	0.15	0.48
IR	−73	0.74
CM	0.35	0.08
**PopLHO**	***r***	***P***
AE	0.06	0.74
ER	−0.12	0.56
ABD	0.13	0.5
IR	−0.42	44
CM	0.02	0.9

Abbreviations: ABD: abduction; AE: active elevation; CMS: Constant-Murley score; DSA: distalisation shoulder angle; ER: external rotation; IR: internal rotation; LSA: lateralisation shoulder angle; popAHD: postoperative acromiohumeral distance; popLHO: postoperative lateral humeral offset; PreAHD: preoperative acromiohumeral distance; preLHO: preoperative lateral humeral offset.

### Linear Regression and Quadratic Component

**Fig. 3:**
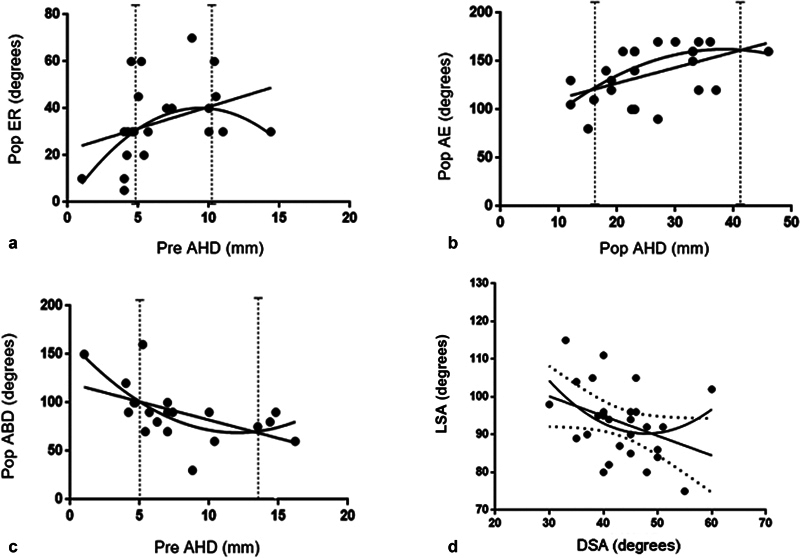
Linear regression and quadratic component. a) between popER and preAHD; b) between pop AE and popAHD; c) between pop ABD and preAHD; d) between LSA and DSA. Pop ER: postoperative external rotation; preAHD: preoperative acromiohumeral distance; popAE: postoperative active elevation; popADH: postoperative acromiohumeral distance; LSA: lateralisation shoulder angle; DSA: distalisation shoulder angle.

Postoperative ER showed a positive linear regression with preoperative AHD (r ^2^ : 0.12). The addition of a quadratic component produced an increase in fit (r ^2^ : 0.3; p: 0.02) and with preoperative AHD values of 5 and 10 mm, the best ER angles were found ( Fig. 3a ).

Postoperative AE showed a positive linear regression with postoperative AHD (r ^2^ : 0.24). The addition of a quadratic component produced a significant increase in fit (r ^2^ : 0.22; p: 0.02) with better postoperative AE angles among 18 and 38 mm of postoperative AHD ( Fig. 3b ).

Postoperative ABD showed an inverse linear regression with preoperative AHD (r ^2^ : 0.19). The addition of a quadratic component produced an increase in fit (r ^2^ : 0.24; p:0.02). The best ABD angles (90-160°) were found with preoperative AHD values between 5.7 and 13.5. ( Fig. 3c ). Finally, there was found a negative Pearson correlation between LSA and DSA (r ^2^ :-0.38; p:0.047) ( Fig. 3d ) In all these cases a statistically significant relationship was found.

No quadratic component was found between the DSA and the AE; neither between LSA and ABD (r2: 0.05; p:0.33), LSA and ER or LSA and IR, nor between postoperative AHD and ABD or postoperative LHO and ABD.

### Area Under the Curve and Predictions

**Fig. 4:**
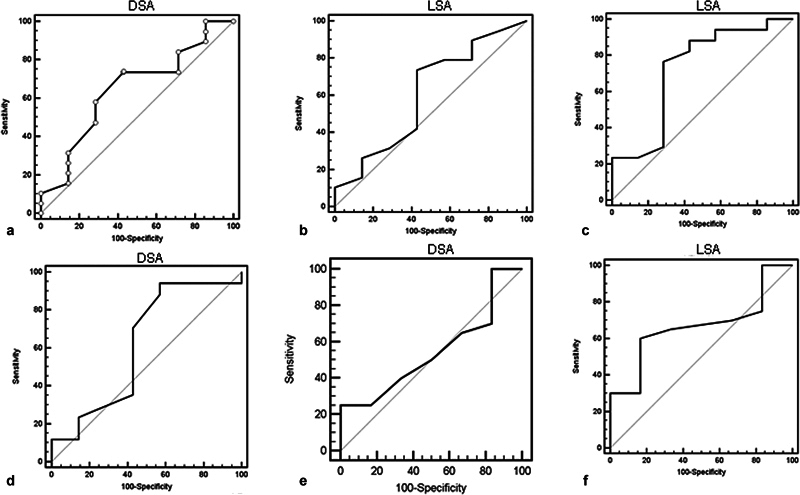
ROC curves. a) DSA and AE >106°; b) LSA and AE >106°; c) LSA and ABD >76°; d) DSA and ABD >76°; e) DSA and ER >20°; f) LSA and ER >20°. DSA: distalisation shoulder angle; AE: active elevation; ABD: abduction; ER: external rotation; LSA: lateralisation shoulder angle.

The area under the ROC curve AUC-ROC was used. A DSA≤45° can predict a postoperative AE > 106°, with a sensibility (SE) of 73.7% (95% IC: 48.8-90.9) and specificity (SP) of 57.1% (95% IC: 29-96.3), with an area under the curve (AUC) of 64% (95% IC: 0.4-0.8. ( Fig. 4a ). A LSA≥86° predicts an AE > 106° with an AUC of 0.6 (95% IC: 0.4-0.8) with a SE of 73.7% (95% IC: 48.8-90.9) and a SP of 57.1 (95% IC: 18-90.1( Fig. 4b ). A LSA > 80° can predict ABD > 76° with 94,12% of SS and 50% of SP with AUC of 0.62 (95% IC: 0.4-0.8) ( Fig. 4c ).

For DSA and ABD > 76° ( Fig. 4d ) and DSA or LSA regarding ER >20° ( Fig. 4e-4f ), the AUC was fair at 0.62, 0,55 and 0.67 respectively, and the models were not statistically significant.

The best AE values (>106°) are found with DSA between 40-45° and with LSA between 80-90°. Lower or higher values have lower PPV ( Tables 4a y 4b ).

**Table 4:** 4a) DSA and AE > 106°; 4b) LSA and AE > 106°; 4c) LSA and ABD > 76°

**4a.**		
**DSA (°)**	**VPP (%)**	**CI 95%**
≤ 35°	72.7	18–99.1
≤ 40°	79.1	45.2–96.7
≤ 45°	80.1	54–95.1
≤ 50°	70.7	48.3–87.6
**4b.**		
**LSA (°)**	**VPP (%)**	**CI 95%**
≤ 80°	74.2	51.4–90.3
≤ 85°	80.6	54–90.1
≤ 90°	77.3	49–94.5
**4c.**		
**LSA (°)**	**VPP (%)**	**CI 95%**
≤ 90°	75.03	39.4–95.6
≤ 95°	84.8	58.4–97.5
≤ 100°	76.64	53.3–92.1

Abbreviations: ABD, abduction; AE, active elevation; CI, confidence interval; DSA, distalisation shoulder angle; LSA, lateralisation shoulder angle; VPP, predictive positive value.

The best ABD values are found with LSA between 90–100°. Values below or greater have lower PPV ( Table 4c ).

## Discussion

The main findings of this study were that in a lateralised RSA with a 135° humeral inclination ranges of AE >106° were found with DSA between 40–45° and LSA of 80–90°, while ABD > 76° was more frequently found with LSA 90–100°.

The first author who proposed the usage of DSA and LSA was Boutsiadis et al., 8 who included two different implant designs of 145° and 155° humeral inclination. Their findings were the existence of a positive linear regression with LSA and EA and a highest EA and ABD with a DSA between 40° and 65°, and the best values of RE with a LSA within 75°-100°. 8 Berthold et al., 5 who also reported in 61 patients a correlation between AE and DSA among 40°-60° and LSA among 75°-95° using a 135° humeral inclination implant. Those results were similar to our study, where better EA was related to 40–45° of DSA and 80–90° of LSA. In our series we found LSA to be associated with better ABD when calculated between 90°-100°. In our knowledge, this is the first time that this relation is described.

We observed that AE > 106° could be predicted with a DSA between 40–45° of and with LSA between 80–90°. Those results can be compared to previous studies, where the lowest AE (<100°) was related to DSA <40° or >70° 5 8 and with a LSA >95°.

As other authors 14 15 16 we didn't find a cut-off DSA or LSA value for RE as Boutsiadis et al. 8 did predicting an ER > 16° with a range of LSA between 75° and 95°. There was a directly proportional association between preoperative AHD, like Berthold described, 5 with the better ranges of ER with distances of 5–10 mm. This radiographic measurement had an inverse association with ABD, finding values of 90–160° with AHD of 5.7 y 13.5 mm. As far as postoperative AHD is concerned, it was directly associated with AE. Its measurements between 18–38 mm were related to better ranges of AE. It was previously mentioned by Jobin et al. 17 who found that patients with >135° of AE had a postoperative AHD > 38 mm in 90% of the cases, and less than 135° with a postoperative AHD < 38 mm in 45% of them. Lädermann et al. 18 described a positive linear relationship between AHD and AE in a computer-based model. Berthold found a significant moderate correlation between RE and postoperative AHD, 5 nevertheless our findings were not similar. To summarise, according to our results, the best ROM could be obtained with LSA between °80 and 100 ° and with a DSA >40° and ≤45°Both LSA and DSA provide an estimation of the lateralisation and distalisation of the humerus after a RSA. Beltrame et al. 15 found a direct relationship between LSA and lateralised RSA and between DSA and more distalised RSA. We identified a negative linear correlation between those angles, as Boutsiadis et al. 8 and Beltrame et al. 15 reported, which means there is a point in which too much distalisation leads to less lateralisation. Lateralisation has been demonstrated to increase postoperative AE and ER, 19 by restoring the anatomic centre of rotation, optimising recruitment of remaining cuff muscle fibres and preserving the rotational moment of the ssc and teres minor, and also increasing the arm's moment of deltoid by 42%. 7 20 EA could be influenced by lateralisation, deltoid's volume and comorbidities of patients 21 even though there is still debate around this topic. 16

Lateralisation of RSA can be generated whether at the glenoid or humeral side, or at both of them. With the BIO-RSA, there is only glenoid lateralisation, 22 in contrast to the reverse Arrow. 23 In this series, LSA was between 80° and 110° for optimal implant lateralisation, as far as ER and ABD is concerned. Caution must be taken with excessive lateralisation due to risk of neuropraxia and acromial fracture. 24 25

Humeral distalisation allows to increase the tension in the deltoid muscle thus increasing AE. 3 It is thought that the optimal humeral lengthening should be around 2cm, 26 however, excessive distalisation of the RSA could generate neurapraxia. 27

Resulting Constant-Murley score was 69.9 ± 7.8, in concordance with other authors, oscillating between 59 points in 45 patients at 40 months' follow-up, and 86 points using a lateralised implant after 10 years of follow-up. 28 29 We did not find any relation between LSA or DSA with postoperative CM, in contrast to Boutsiadis et al., 8 who described a significant association between CM in the mobility section and LSA.

Limitations of our retrospective study were that the intraoperative status of the subscapularis was not documented in all cases. Also, the size of the glenosphere may interfere with the ROM 30 which was not taken into account in this study. Another limitation was the small number of evaluated patients and the fact that radiographies, even if standardised, can show variances depending on patients positioning during imaging.

## Conclusions

In this study we found that a DSA between 40–45° and a LSA of 80–100° could lead to better range of motion regarding AE and ABD in patients with rotator cuff arthropathy treated with RSA.
