# Supplementary material for: Can Distalisation and Lateralisation Shoulder Angles in Reverse Arthroplasty Interfere with the Functional Results in Patients with Rotator Cuff Arthropathy?
Source: Rev Bras Ortop (Sao Paulo). 2024 Mar 21;59(1):e93–e100. doi: 10.1055/s-0044-1779609 (PMC10957274; doi:10.1055/s-0044-1779609)
Supplement: Related texts

# Research article: Os ângulos de distalização e lateralização do ombro na artroplastia reversa podem interferir nos resultados funcionais em pacientes com artropatia do manguito rotador?

**Keywords:** artroplastia, manguito rotador, ombro

## Abstract

**Objetivo:** Avaliar a influência dos valores radiográficos nos resultados clínicos e funcionais em pacientes tratados com artroplastia reversa para artropatia do manguito rotador utilizando um desenho lateralizado.

**Métodos:** Foi realizada uma análise retrospectiva. Foram registradas as demografias dos pacientes, bem como o intervalo de movimento pré-operatório e pós-operatório. A função foi calculada usando a pontuação Constant-Murley tanto antes como depois do procedimento. Radiografias pré e pós-operatórias anteroposteriores e axiais do ombro afetado foram analisadas. Nas imagens pré-operatórias, foram calculados a distância acromioumeral (DAU) e *offset* lateral umeral (OLU). As medidas pós-operatórias incluídas foram DAU, OLU, ângulo de distalização do ombro (ADO) e ângulo de lateralização do ombro (ALO). Foi realizada uma análise de regressão linear e regressão quadrática para determinar seu grau de associação com os resultados funcionais finais. Aplicando uma análise de regressão quadrática e curvas ROC, os valores de corte foram determinados em relação aos ângulos acima mencionados e o valor preditivo positivo foi calculado.

**Resultados:** As maiores faixas de elevação anterior (EA) foram encontradas com ADO entre 40 e 45° e ALO entre 80 e 90°, enquanto a melhor abdução (ABD) foi observada com ALO de 90 e 100°. A DAU pré-operatória foi correlacionada com a rotação externa (RE) (r _s_ : 0,47; p: 0,049). A DAU pós-operatória apresentou uma relação diretamente proporcional com a EA (r _s_ : 0,49; p: 0,03). A ABD pós-operatória mostrou uma regressão linear inversa com a DAU pré-operatória (r _s_ : -0,44, p: 0,047). O ALO e o ADO estavam inversamente relacionados.

**Conclusão:** Determinamos que um ADO entre 40 e 45° e um ALO de 80 e 100° poderia levar a uma melhor amplitude de movimento em relação à EA e ABD em pacientes com artropatia do manguito rotador tratados com artroplastia reversa de ombro.

## Introdução

O desenho original da artroplastia invertida (AI) do ombro por Grammont em 1985 para o tratamento da artropatia do manguito rotador (AMR) consistia em medialização e distalização do centro de rotação da articulação glenoumeral para melhorar o braço de alavanca deltoide e, assim, aumentar a amplitude da elevação anterior (EA) e a abdução (ABD). 1 2 3 Esses implantes foram associados a algumas complicações específicas, como geração de incisuras escapulares e danos neurológicos 1 2 3 que diminuíram graças à introdução de artroplastias reversas do ombro (ARO) lateralizadas, também diminuindo a incidência de luxação protética e aumentando a amplitude de rotação. 4

Há controvérsias em relação ao posicionamento ideal do implante para maximizar a amplitude de movimento (ADM) e reduzir o risco de complicações. Debate-se também os valores radiográficos e sua influência nos desfechos pós-operatórios. 5 Vários autores tentam determinar o grau ideal de lateralização e distalização na ARO, embora os métodos tenham sido considerados muito exigentes para a prática clínica diária. 5 6 7 8 Os ângulos de distalização e lateralização do ombro descritos por Boutsiadis et al. 8 acerca da posição do úmero em relação à escápula, poderiam representar ferramentas reprodutíveis e determinantes dos desfechos clínicos.

O objetivo deste estudo foi identificar os valores radiográficos e avaliar sua influência nos resultados em pacientes tratados com ARO para AMR utilizando um desenho lateralizado com 135° de inclinação umeral.

## Métodos

Este estudo é uma análise retrospectiva de casos atendidos entre janeiro de 2018 e janeiro de 2020 e foi aprovado pelo Comitê de Ética.

## Seleção de Pacientes

Os critérios de exclusão foram pacientes tratados com próteses medializadas ou com inclinação umeral de 145° ou 155°; presença concomitante de fraturas da cabeça do úmero ou da glenoide; ausência de radiografias pré-operatórias; cirurgias de revisão; acompanhamento insuficiente e doenças neuromusculares. Os pacientes incluídos tiveram acompanhamento mínimo de 12 meses e foram submetidos ao tratamento da ARM (Hamada ≥ 3) com prótese lateralizada e inclinação umeral de 135°. Esses indivíduos tinham 18 anos ou mais e deltoide funcional.

## Técnica Cirúrgica

A prótese lateralizada Arrow® (FH Orthopaedics, Mulhouse, França) foi utilizada.

Com o paciente em posição de cadeira de praia e anestesia do plexo, a abordagem deltopeitoral foi realizada com tenotomia insercional completa do subescapular e secção da cápsula articular. A cabeça do úmero foi luxada em rotação externa máxima e a osteotomia do úmero foi realizada com 20° de retroversão e 135° de inclinação. Posteriormente, o canal umeral foi submetido a raspagens crescentes até a obtenção de uma sensação de atrito cortical. A lima de maior calibre foi mantida no local para proteger o úmero proximal durante o procedimento na glenoide.

Ao realizar a exposição da glenoide, os osteófitos e o lábio degenerativo foram ressectados para otimizar a visualização da superfície articular. Em seguida, a cartilagem articular foi fresada, preservando o máximo de estoque ósseo possível. A implantação de metaglene foi feita projetando uma inclinação inferior de 10° com versão neutra. A fixação definitiva utilizou dois parafusos de compressão de 5,5 mm. Este sistema permitiu a utilização de glenosferas de 36, 39 ou 42 mm.

O componente umeral definitivo foi colocado não cimentado desde que a qualidade óssea metafisária o permitisse. A seguir, o tamanho do inserto de polietileno final foi escolhido. Assim como o tamanho da glenosfera, estes parâmetros foram decididos de acordo com a tensão deltoide intraoperatória e a estabilidade do implante.

Todos os pacientes foram submetidos à imobilização por 30 dias com tipoia de tipo Velpeau e iniciaram exercícios de mobilidade passiva uma semana após a cirurgia e reabilitação quatro semanas depois.

## Avaliação Clínica

As ADMs pré-operatória e pós-operatória no último acompanhamento foram determinadas. A mobilidade ativa foi avaliada em graus, utilizando goniômetro, em EA, RE1, ABD e rotação interna (RI) com a mão nas costas, registrando o segmento alcançado pelo polegar como nível máximo conforme a descrição de Greene e Heckman 9 e pontuado de acordo com Levy et al. 10

A função do deltoide foi definida pelo cirurgião responsável pelo tratamento, de acordo com a escala motora de Daniel, considerando o funcionamento do deltoide ao igualar ao valor de M5. 11

A função foi calculada utilizando o escore de Constant-Murley adaptado para a população argentina. 12

## Avaliação Radiológica

Foram utilizadas radiografias anteroposterior (AP) e axial do ombro afetado, tanto pré-operatórias quanto pós-operatórias. Considerando a importância do posicionamento correto e da técnica radiológica, todas as imagens foram realizadas pela mesma equipe e o mesmo fluoroscópio.

As medidas foram determinadas na projeção AP por dois pesquisadores que desconheciam os desfechos clínicos com o software Synapse 3d® (Fujifilm Healthcare®), considerando a média interobservador. A concordância interobservador foi calculada pelo coeficiente de correlação intraclasse (CCI). O poder do CCI foi considerado quando a concordância foi superior a 0,8. O CCI foi quase perfeito em todas as medições, como demonstrado na Tabela 1 .

**Tabela 1:** Valores radiográficos expressos como média e desvio-padrão (DP)

	Média (DP)	Intervalo	CCI	IC de 95%
**Idade (anos)**	72 (±7,1)	61–84		
**DAU pré-operatória**	7,27 mm (±4,1)	1–16,2	0,96	(0,81–0,99)
**OLU pré-operatório**	12,56 mm (±5,8)	3–23,5	0,98	(0,95–0,99)
**DAU pós-operatória**	25,6 mm (±8,3)	12–46	0,97	(0,93–0,98)
**OLU pós-operatório**	18,6 mm (±7,3)	2–28,2	0,95	(0,89–0,97)
**ADO**	43,2° (±6,8)	30–60	0,88	(0,53–0,96)
**ALO**	92,5° (±10,1)	80–115	0,82	(0,62–0,92)
**CM**	69,9 (±7,8)	47–83		

A tabela mostra o coeficiente de correlação intraclasse de cada medida e seu intervalo.

Abreviações: IC, intervalo de confiança; CM, escore de Constant-Murley; ADO, ângulo de distalização do ombro; ICC, coeficiente de correlação intraclasse; ALO, ângulo de lateralização do ombro; DAU, distância acromioumeral; OLU, *offset* lateral do úmero; DAU, distância acromioumeral; OLU, *offset* lateral do úmero.

Nas imagens pré-operatórias, foram calculados os seguintes parâmetros: distância acromioumeral (DAU) e *offset* lateral do úmero (OLU). As medidas pós-operatórias incluíram DAU, OLU, ADO e ALO ( Fig. 1 ).

- O ALO ( Fig. 1A ) foi determinado com base em três pontos de referência: a borda superior do tubérculo glenoidal, a borda mais lateral do acrômio e a borda mais lateral do tubérculo maior. Uma linha uniu o tubérculo glenoidal superior à borda mais lateral do acrômio. Uma segunda linha conectou este último ponto com a borda lateral da tuberosidade maior. O ângulo entre essas duas linhas corresponde ao ALO.
- O ADO ( Fig. 1B ) considerou a borda superior do tubérculo glenoidal, a borda mais lateral do acrômio e a borda mais superior do tubérculo maior. O ADO foi calculado traçando-se uma linha da borda mais lateral do acrômio até o tubérculo glenoidal superior e, daí, outra linha até a borda mais superior da tuberosidade maior. O ângulo entre essas duas linhas correspondeu ao ADO.
- A DAU foi medida calculando a distância perpendicular entre a porção mais lateral do acrômio e uma linha paralela à borda superior da tuberosidade maior ( Fig. 1C-D ).
- O OLU foi calculado com base na distância da linha da DAU até a projeção da borda mais lateral da tuberosidade maior ( Fig. 1E-F ).

**Fig. 1:** Descrições das medidas. a) ângulo de lateralização do ombro; b) ângulo de distalização do ombro; c) distância acromioumeral pré-operatória; d): distância acromioumeral pós-operatória; e) deslocamento lateral do úmero pré-operatório; f) *offset* lateral do úmero pós-operatório.

A classificação de Hamada e Fukuda 13 determinou o grau de osteoartrite glenoumeral.

## Análise Estatística

As variáveis descritivas contínuas foram expressas como média, desvio-padrão e amplitude. As variáveis qualitativas foram expressas em porcentagens. Uma análise de regressão linear determinou o grau de associação entre os ângulos pré-operatórios e pós-operatórios e a mobilidade. Uma análise de regressão quadrática foi realizada e a área sob a curva (AUC, do inglês *area under the curve* ) característica de operação do receptor (ROC, do inglês, *receiver operating characteristic* ) (AUC-ROC) foi calculada. O valor do ponto de corte foi utilizado como o valor cuja sensibilidade e especificidade estavam mais próximas do valor da AUC-ROC e a diferença absoluta entre o valor de sensibilidade e especificidade foi mínima. Os valores de corte foram determinados em relação aos ângulos ADO e ALO. Os programas Graph Pad Prism 8.02 e MedCalc12.0 foram empregados para análise.

## Resultados

## Pacientes

Dos 57 pacientes submetidos à ARO, 35 foram diagnosticados com ARM. Oito indivíduos foram excluídos; não foi possível obter as radiografias de três pacientes, quatro não tiveram acompanhamento suficiente e os demais receberam próteses medializadas ( Fig. 2 ). O estudo incluiu 27 pacientes com idade média de 72,0 ± 7,1 anos. (77,7% mulheres, n: 21, sendo 20 ombros direitos). A ADM não foi analisada em relação ao tamanho da glenosfera, que foi de 36 mm em 24 casos e 39 mm em três casos. O tempo de acompanhamento foi de 19,3 ± 6,9 meses após a cirurgia. O ALO médio foi de 92,5° (85 a 115°) e o ADO médio foi de 43,2° (30 a 60°). Os valores médios das medidas radiográficas estão descritos na Tabela 1 , assim como o desvio-padrão, o CCI e os intervalos de confiança (CI) dos coeficientes. A Tabela 2 mostra a mobilidade pré e pós-operatória e as diferenças significativas entre os valores, exceto de ABD (p: 0,56)

**Fig. 2:** Fluxograma de pacientes incluídos e excluídos. ARO: artroplastia reversa do ombro; ALM; artropatia da laceração de manguito.

**Tabela 2:** Valores pré e pós-operatórios de mobilidade

	Média (DP) Pré-operatória	Média (DP) Pós-operatória)	p
**Elevação anterior**	90,21° (39,8°)	131,2° (32°)	0,0002
**Rotação externa**	12,3° (19,2)	35,2° (16,8°)	<0,0001
**Rotação interna**	L4-L5 (3,8) SD 2,2	L1-L3 (5,5) SD 2,4	0,013
**Abdução**	68,7° (31,9°)	87,1° (28,2°)	0,56

## Análise de Correlação

Houve uma associação diretamente proporcional entre o DAU pré-operatório e RE e a rotação interna (RI) (r _s_ : 0,47 e r _s_ : 0,44, respectivamente) e uma correlação negativa de Pearson com a ABD (r _s_ : -0,44). A DAU e a EA pós-operatória apresentaram associação de Spearman diretamente proporcional (r _s_ : 0,49). A Tabela 3 revela a ausência de associação entre os demais ângulos estudados.

**Tabela 3:** Análise de correlação

DAU pré-operatória	*r*	*p*
EA	0,0004	0,99
RE	0,47	0,049
ABD	−0,44	0,047
RI	0,44	0,046
CM	0,36	0,07
**OLU pré-operatório**	***r***	***p***
EA	−0,0025	0,9
RE	0,17	0,42
ABD	−0,04	0,86
RI	−0,43	0,04
CM	0,12	0,6
**DAU**	***r***	***p***
EA	0,13	0,5
RE	−0,05	0,8
ABD	25	0,9
RI	0,06	0,8
CM	0,06	0,7
**ALH**	***r***	***p***
EA	−8	0,7
RE	−0,14	0,49
ABD	0,25	0,2
RI	−0,24	0,28
CM	−0,3	0,13
**DAU pós-operatória**	***r***	***P***
EA	0,49	0,03
RE	0,32	0,11
ABD	0,15	0,48
RI	−73	0,74
CM	0,35	0,08
**OLU pós-operatório**	***r***	***P***
EA	0,06	0,74
RE	−0,12	0,56
ABD	0,13	0,5
RI	−0,42	44
CM	0,02	0,9

Abreviações: ABD: abdução; EA: elevação ativa; CM: escore de Constant-Murley; ADO: ângulo de distalização do ombro; RE: rotação externa; RI: rotação interna; ALO: ângulo de lateralização do ombro; DAU: distância acromioumeral; OLU: *offset* lateral do úmero.

## Regressão Linear e Componente Quadrático

A RE pós-operatória apresentou regressão linear positiva com a DAU pré-operatória (r ^2^ : 0,12). A adição de um componente quadrático aumentou o ajuste (r ^2^ : 0,3; p: 0,02). Além disso, os valores pré-operatórios de DAU de 5 e 10 mm melhoraram os melhores ângulos de RE ( Fig. 3a ).

**Fig. 3:** Regressão linear e componente quadrática. a) entre RE pós-operatório e DAU pré-operatória; b) entre EA pós-operatória e DAU pós-operatória; c) entre ABD pós-operatória e DAU pré-operatória; d) entre ALO e ADO. RE: rotação externa; DAU: distância acromioumeral; EA elevação ativa; ALO: ângulo de lateralização do ombro; ADO: ângulo de distalização do ombro.

A EA pós-operatória apresentou regressão linear positiva com a DAU pós-operatória (r ^2^ : 0,24). A adição de um componente quadrático produziu um aumento significativo no ajuste (r ^2^ : 0,22; p: 0,02) com melhores ângulos pós-operatórios de EA entre 18 e 38 mm de DAU pós-operatória ( Fig. 3b ).

A ABD pós-operatória apresentou regressão linear inversa com a DAU pré-operatória (r ^2^ : 0,19). A adição de um componente quadrático aumentou o ajuste (r ^2^ : 0,24; p: 0,02). Os melhores ângulos de ABD (90 a 160°) foram associados a valores pré-operatórios de DAU entre 5,7 e 13,5 ( Fig. 3c ). Por fim, a correlação de Pearson entre ALO e ADO foi negativa (r ^2^ :-0,38; p: 0,047) ( Fig. 3d ). Todos estes casos apresentaram relação estatisticamente significativa.

Não houve nenhum componente quadrático entre a ADO e a EA ou entre ALO e ABD (r ^2^ : 0,05; p: 0,33), ALO e RE ou ALO e RI, nem entre DAU pós-operatória e ABD ou OLU pós-operatório e ABD.

## Área Sob a Curva e Previsões

A AUC-ROC foi calculada. O ADO ≤ 45° pode prever uma EA pós-operatória > 106° com sensibilidade (SE) de 73,7% (IC de 95%: 48,8 a 90,9) e especificidade (ESP) de 57,1% (IC de 95%: 29 a 96,3), com AUC de 64% (IC de 95%: 0,4 a 0,8) ( Fig. 4a ). O ALO ≥ 86° prevê uma EA > 106° com AUC de 0,6 (IC de 95%: 0,4 a 0,8), SE de 73,7% (IC de 95%: 48,8 a 90,9) e ESP de 57,1 (IC de 95%: 18 a 90,1) ( Fig. 4b ). O ALO > 80° pode prever ABD > 76° com 94,12% de SE, 50% de ESP e AUC de 0,62 (IC de 95%: 0,4-0,8) ( Fig. 4c ).

**Fig. 4:** Curvas ROC. a) ADO e EA >106°; b) ALO e EA >106°; c) ALO e ABD >76°; d) ADO e ABD >76°; e) ADO e RE >20°; f) ALO e RE >20°. ROC: característica de operação do receptor; ASD: ângulo de distalização do ombro; EA: elevação ativa; ABD: abdução; RE: rotação externa; ALO: ângulo de lateralização do ombro.

Com ALO e ABD >76° ( Fig. 4d ) e ADO ou ALO referente à RE >20° ( Fig. 4e-4f ), a AUC foi razoável, de 0,62, 0,55 e 0,67, respectivamente, e os modelos não foram estatisticamente significativos.

Os melhores valores de EA (>106°) foram encontrados com ADO entre 40 e 45° e ALO entre 80 e 90°. Valores menores ou maiores têm valor preditivo positivo (VPP) menor ( Tabelas 4a e 4b ).

**Tabela 4:** 4a) ADO e EA > 106°; 4b) ALO e EA > 106°; 4c) ALO e ABD > 76°

**4a.**		
**ADO (°)**	**VPP (%)**	**IC de 95%**
≤ 35°	72,7	18–99,1
≤ 40°	79,1	45,2–96,7
≤ 45°	80,1	54–95,1
≤ 50°	70,7	48,3–87,6
**4b.**		
**ALO (°)**	**VPP (%)**	**IC de 95%**
≤ 80°	74,2	51,4–90,3
≤ 85°	80,6	54–90,1
≤ 90°	77,3	49–94,5
**4c.**		
**ALO (°)**	**VPP (%)**	**IC de 95%**
≤ 90°	75,03	39,4–95,6
≤ 95°	84,8	58,4–97,5
≤ 100°	76,64	53,3–92,1

Abreviações: ABD, abdução; EA, elevação ativa; IC, intervalo de confiança; ADO, ângulo de distalização do ombro; ALO, ângulo de lateralização do ombro; VPP, valor preditivo positivo.

Os melhores valores de ABD foram associados ao ALO entre 90 e 100°. Valores menores ou maiores apresentaram VPP menor ( Tabela 4c ).

## Discussão

Os principais achados deste estudo foram que um ARO lateralizado com inclinação umeral de 135° apresentou faixas de EA >106°, ADO entre 40 e 45° e ALO entre 80 e 90°, enquanto a ABD >76° foi mais observada com ALO entre 90 e 100°.

Os primeiros autores a proporem o uso de ADO e ALO foram Boutsiadis et al., 8 que incluíram dois desenhos diferentes de implantes com inclinação umeral de 145° e 155°. Estes autores observaram a existência de regressão linear positiva com ALO e EA e maior EA e ABD com ADO entre 40° e 65° e melhores valores de RE com ALO entre 75° e 100°. 8 Berthold et al. 5 também relataram uma correlação entre EA e ADO entre 40° e 60° e ALO entre 75° e 95° utilizando implante de inclinação umeral de 135° em 61 pacientes. Esses resultados foram semelhantes ao nosso estudo, em que uma melhor EA foi relacionada a 40 a 45° de ADO e 80 a 90° de ALO. Nossa série revelou que o ALO está associado à melhor ABD quando calculado entre 90 e 100°. Até onde temos conhecimento, esta é a primeira vez que esta relação é descrita.

Observamos que a EA >106° poderia ser prevista quando ADO era entre 40 e 45° e ALO entre 80 e 90°. Esses resultados podem ser comparados a estudos anteriores, onde a menor EA (<100°) foi relacionada ao ADO <40° ou >70° 5 8 e ALO >95°.

Como outros autores, 14 15 16 não encontramos um valor de corte de ADO ou ALO relacionado à RE, ao contrário de Boutsiadis et al., 8 que previram RE > 16° quando ALO variava entre 75 e 95°. Houve uma associação diretamente proporcional entre DAU pré-operatória, conforme descrito por Berthold, 5 e os melhores intervalos de RE com distâncias entre 5 e 10 mm. Essa medida radiográfica teve associação inversa com ABD, que apresentou valores entre 90 e 160° com DAU de 5,7 e 13,5 mm. A DAU pós-operatória foi diretamente associada à EA. As medidas entre 18 e 38 mm foram relacionadas a melhores faixas de EA. Isso foi mencionado por Jobin et al., 17 que verificaram que pacientes com EA >135° apresentavam DAU pós-operatória >38 mm em 90% dos casos e, em 45% dos indivíduos, a EA era inferior a 135° quando a DAU pós-operatória era < 38 mm. Lädermann et al. 18 descreveram uma relação linear positiva entre DAU e EA em um modelo computacional. Berthold observou uma correlação moderada e significativa entre RE e DAU pós-operatória, 5 mas nossos achados não foram semelhantes. Resumindo, de acordo com nossos resultados, a melhor ADM pode ser obtida com ALO entre 80 e 100° e ADO >40° e ≤45°. Tanto ALO quanto ADO permitiram uma estimativa da lateralização e distalização do úmero após a ARO. Beltrame et al. 15 encontraram uma relação direta entre ALO e ARO lateralizado e entre ASD e ARO mais distalizado. Identificamos uma correlação linear negativa entre esses ângulos, como relataram Boutsiadis et al. 8 e Beltrame et al. 15 , o que significa que há um ponto em que muita distalização leva a menos lateralização. Demonstrou-se que a lateralização aumenta EA e RE pós-operatórias, 19 restaurando o centro anatômico de rotação, otimizando o recrutamento das fibras musculares restantes do manguito e preservando o momento rotacional do subescapular e redondo menor, também aumentando o momento do deltoide em 42%. 7 20 A EA pode ser influenciada pela lateralização, volume do deltoide e comorbidades dos pacientes, 21 embora este tópico ainda seja debatido. 16

A lateralização do ARO pode ser gerada no lado glenoide e/ou umeral. O BIO-LSA causa apenas lateralização da glenoide, 22 em contraste com o Arrow reverso. 23 Nesta série, o ALO ficou entre 80 e 110° para lateralização ideal do implante no que diz respeito à RE e ABD. Deve-se ter cuidado com lateralização excessiva devido ao risco de neuropraxia e fratura acromial. 24 25

A distalização do úmero permite aumentar a tensão no músculo deltoide, elevando assim a EA. 3 Acredita-se que o alongamento ideal do úmero deve ser em torno de 2cm, 26 mas a distalização excessiva do ARO pode gerar neurapraxia. 27

O escore de Constant-Murley foi de 69,9 ± 7,8, em concordância com outros autores, oscilando entre 59 pontos em 45 pacientes aos 40 meses de acompanhamento e sendo de 86 pontos com implante lateralizado após 10 anos de acompanhamento. 28 29 Não encontraram relação entre ALO ou ADO e o escore de Constant-Murley pós-operatório, ao contrário de Boutsiadis et al., 8 que descreveram uma associação significativa entre a seção de mobilidade do escore e ALO.

As limitações do nosso estudo retrospectivo foram a ausência de documentação do *status* intraoperatório do subescapular em todos os casos. Além disso, o tamanho da glenosfera poderia interferir na ADM, 30 que não foi levada considerada neste estudo. Outra limitação foi o pequeno número de pacientes avaliados e o fato de que as radiografias, mesmo padronizadas, poderem apresentar variações dependendo do posicionamento do paciente durante o exame.

## Conclusões

Este estudo revela que o ADO entre 40 e 45° e o ALO entre 80 e 100° poderia melhorar a amplitude de movimento em relação à EA e ABD em pacientes com artropatia do manguito rotador submetidos à ARO.
